# Metabolic syndrome does not influence the phenotype of *LRRK2* and *GBA* related Parkinson’s disease

**DOI:** 10.1038/s41598-020-66319-9

**Published:** 2020-06-09

**Authors:** Avner Thaler, Shani Shenhar-Tsarfaty, Yanay Shaked, Tanya Gurevich, Nurit Omer, Anat Bar-Shira, Mali Gana-Weisz, Orly Goldstein, Meir Kestenbaum, Jesse M. Cedarbaum, Avi Orr-Urtreger, Nir Giladi, Anat Mirelman

**Affiliations:** 1 Movement Disorders Unit, Neurological Institute, Tel-Aviv Medical Center, Tel Aviv-Yafo, Israel; 2 Sackler School of Medicine, Tel-Aviv University, Tel Aviv-Yafo, Israel; 3 Sagol School of Neuroscience, Tel-Aviv University, Tel Aviv-Yafo, Israel; 4 Department of Internal Medicine “C”, “D”, and “E”, Tel-Aviv Medical Center, Tel Aviv-Yafo, Israel; 5 Genetic Institute, Tel-Aviv Medical Center, Tel Aviv-Yafo, Israel; 6 Genomic Research Laboratory for Neurodegeneration, Tel-Aviv Medical Center, Tel Aviv-Yafo, Israel; 7 Neurology department, Meir Hospital, Kfar-Saba, Israel; 8 Biogen Inc, Cambridge, MA USA; 9 Coeruleus Clinical Sciences LLC, Woodbridge, CT USA; 10 Laboratory of Early Markers of Neurodegeneration, Neurological Institute, Tel-Aviv Medical Center, Tel Aviv-Yafo, Israel

**Keywords:** Genetics, Neuroscience

## Abstract

In order toevaluate the influence of the metabolic syndrome (MS) (obesity, hypertension, elevated triglycerides, reduced levels of HDL cholesterol and glucose impairment) on the phenotype of *LRRK2* and *GBA* Parkinson’s disease (PD), and on the prevalence of prodromal features among individuals at risk, we collected, laboratory test results, blood pressure, demographic, cognitive, motor, olfactory and affective information enabling the assessment of each component of MS and the construction of the MDS prodromal probability score. The number of metabolic components and their levels were compared between participants who were separated based on disease state and genetic status. One hundred and four idiopathic PD, 40 *LRRK2*-PD, 70 *GBA*-PD, 196 healthy non-carriers, 55 *LRRK2*-NMC and 97 *GBA*-NMC participated in this study. PD groups and non manifesting carriers (NMC) did not differ in the number of metabolic components (p = 0.101, p = 0.685, respectively). *LRRK2*-PD had higher levels of triglycerides (p = 0.015) and higher rates of prediabetes (p = 0.004), while *LRRK2*-NMC had higher triglyceride levels (p = 0.014). NMC with probability rates for prodromal PD above 50% had higher frequencies of hypertriglyceridemia and prediabetes (p < 0.005, p = 0.023 respectively). While elevated triglycerides and prediabetes were more frequent among *LRRK2* carriers, MS does not seem to influence *GBA* and *LRRK2*-PD phenotype.

## Introduction

Parkinson’s disease (PD) is clinically diagnosed based on cardinal motor features with variability related to age of onset, disease progression, response to levodopa and non-motor profile. In recent years a better understanding of the prodromal phase of PD has emerged enabling the construct of the Movement Disorders task force research criteria for prodromal PD^1^. Among Jews from Ashkenazi (AJ) decent, mutations in the glucocerebrosidase (*GBA*) and Leucine Rich Repeat Kinase 2 (*LRRK2*) genes are present among more than 1/3 of the PD population^2^ with specific phenotype for each mutation group^3,4^. Penetrance estimations for these mutations are far from complete^5,6^ due to genetic polymorphisms, environmental causes as well as inflammatory mechanisms^7,8^.

Metabolic Syndrome (MS) is defined as the presence of three out of five components: abdominal obesity or elevated body mass index (BMI), elevated serum triglycerides, low serum HDL, high blood pressure and prediabetes (drug treatment for any of the last four conditions also fulfills the criteria)^9–11^. MS is significantly related to atherosclerosis and vascular cerebral disease^12^ and to neurodegeneration^13^, however its influence on PD clinical phenotype, progression and risk for future development of the disease is still unclear. While Leehey *et al*. detected a faster progression in motor symptoms among PD patients who had MS^14^, the association between MS and risk for PD is conflicting; with one cohort study indicating lower risk^15^, and another pointing towards increased risk^16^.

The association between the components of MS and PD is not clear either. Diabetes mellitus has been suggested to represent a risk for PD but this remains equivocal^17,18^. Possible mechanisms connecting the two states include neuroinflammation, mitochondrial dysfunction and increased oxidative stress^19^. Higher levels of triglycerides and LDL were found to be associated with lower risk for PD^20^. The role of hypertension in PD is conflicting as well^21,22^. These discrepancies result from the choice of population, sample size, comorbid diseases, follow-up periods, analytical techniques and statistical power.

Mutations in the *GBA* gene influence the accumulation of ceramide, a sphingolipid which participates in cellular signaling. The accumulation of ceramide impairs insulin action and promotes apoptosis potentially linking insulin resistance and inflammation^23^ However, to date the relationship between these factors and PD have not been studied.

In order to better characterize the factors that influence the specific phenotypes associated with *GBA* and *LRRK2* PD as well as those that might contribute to disease risk, we assessed the prevalence of MS and its’ different components among genetically determined patients with PD and non-manifesting carriers of mutations in the *LRRK2* or *GBA* genes (NMC) and correlated PD phenotype and future probability for developing PD with the presence of MS. We hypothesized that PD patients with MS would have worse motor and cognitive phenotypes due to comorbidity burden with potential vascular and inflammatory implications, specifically among *GBA*-PD, and that an increase of metabolic components burden would be associated with higher MDS probability scores for developing PD through similar mechanisms.

## Results

A total of 562 subjects participated in this study. Data of 104 iPD patients, 40 *LRRK2*-PD and 70 *GBA*-PD patients is presented in Tables 1 and 2.

**Table 1:** PD cohort characteristics.

	iPD	*LRRK2*-PD	*GBA*-PD	significance
#	104	40	70	
Age(y)	66.61 (10.43)	64.82 (10.03)	64.30 (9.78)	0.302
Disease duration (y)	2.55 (1.98)^@^	3.64 (2.52)	3.98 (2.65)	0.001
Sex m/f	71/33	24/16	46/24	0.644
LEDD	277.11 (279.11)^@^	432.67 (398.41)	468.16 (428.85)	0.002
Orthostatic Hypotension (%)	17.30%	10.00%	22.85%	0.233
MDS-UPDRS	38.14 (18.41)	35.35 (16.70)	43.77 (21.89)	0.077
MoCA	23.86 (3.75)	24.53 (4.44)	23.54 (3.85)	0.455
UPSIT	17.12 (9.87)	20.83 (9.47)^#^	14.51 (9.31)	0.008
RBDQ	3.06 (2.56)	3.08 (2.38)	3.95 (3.53)	0.291
NMSQ	7.46 (4.62)	6.74 (3.78)	8.55 (5.45)	0.083
SCOPA-AUT	16.93 (10.09)	18.03 (11.74)	17.11 (10.85)	0.478
BDI	8.00 (6.13)	6.93 (5.50)	9.31 (7.35)	0.169

iPD – idiopathic Parkinson’s Disease, m-male, f-female, LEDD- LevoDopa Equivalent Daily Dose, MDS-UPDRS- Movement Disorder Society – Unified Parkinson’s Disease Rating Scale, MoCA – Montreal Cognitive Assessment, UPSIT- University of Pennsylvania Smell Identification Test, RBDQ- REM sleep Behavior Disorder Questionnaire, NMSQ- Non-Motor Symptoms questionnaire, SCOPA-AUT- Scale of Outcome in Parkinson’s Disease – Autonomic Dysfunction, BDI- Beck Depression Inventory.

^@^Differences between iPD and *LRRK2*-PD and *GBA*-PD, ^#^Differences between *LRRK2*-PD and GBA-PD

**Table 2:** PD cohort Metabolic Syndrome components.

	iPD	*LRRK2*-PD	*GBA*-PD	significance
#	104	40	70	
HDL (mg/Dl)	56.20 (16.24)	62.02 (19.36)	54.24 (16.35)	0.073
Triglycerides (mg/Dl)	117.24 (57.96)	158.10 (100.48) @	130.90 (60.60)	0.015*
HbA1c (%)	5.65 (0.54)	5.82 (0.51)	5.61 (0.59)	0.108
BMI (kg/m^2^)	25.58 (3.08)	25.77 (3.96)	26.03 (4.16)	0.725
Hypo-HDL (%)	20.00%	7.50%	20.80%	0.160
Hypertriglyceridemia (%)	41.90%	52.50%	38.90%	0.364
Prediabetes (%)	34.30%	65.00%^@^	43.10%	0.004*
Hypertension (%)	75.20%	80.00%	79.20%	0.754
Obesity (BMI > 30) (%)	9.50%	15.00%	19.40%	0.167

iPD – idiopathic Parkinson’s Disease, HDL- High Density Lipoprotein, HbA1c – Hemoglobin A1C, BMI- Body Mass Index. ^*^Significant after correcting for multiple comparisons with Bonferroni adjustment.

^@^Differences between *LRRK2*-PD and *GBA*-PD, iPD.

Both *GBA*-PD and *LRRK2*-PD had longer disease duration and higher LEDD compared with iPD (p < 0.001 and p < 0.002, respectively). *LRRK2*-PD had higher UPSIT scores (p = 0.008), higher rates of prediabetes (p = 0.004) and higher triglyceride levels (p = 0.015) which were correlated with disease duration (r = 0.332, p = 0.036) and LEDD (r = 0.432, p < 0.001).

Groups did not differ in the mean number of metabolic components (p = 0.101), nor in the frequency of MS (presence of any three components as cutoff) (p = 0.211). In the linear regression model, which was constructed to estimate the relationship between MS and its’ components (as dependent variables) and genotype, sex, age, RBDQ, UPDRS-III, MoCA, UPSIT, disease duration, LEDD and NMSQ (independent variables), the number of MS components was associated with age and iPD status (Table 3). There was no association between patients’ characteristics, obesity or low HDL levels. Prediabetes was associated with age and iPD status which accounted for 45.8% of the variance. Hypertension was associated with age and *LRRK2*-PD status, which accounted for 34.9% of the variance. Hypertriglyceridemia was associated with age and the score on the RDBQ questionnaire accounting for 44.8% of the variance.

**Table 3:** Linear regression Models for metabolic factors in PD. B(95%CI) and p values are presented for measures found significant in the model.

	Number of metabolic factors	Prediabetes	Hypertension	hypertriglyceridemia	Low HDL	Obesity
Model	F = 4.928; p < 0.0001	F = 4.068; p < 0.0001	F = 2.121; p = 0.026	F = 3.664; p < 0.0001	F = 0.838; p = 0.593	F = 1.299; p = 0.236
Genotype	0.21(0.03–0.39); 0.02*	0.095(0.009–0.180); 0.031*	0.08(0.008–0.165); 0.032^			
Sex			−0.14(−0.281–0); 0.051			
Age	0.46(0.03–0.06); 0.0001	0.022(0.014–0.030); 0.0001	0.011(0.003–0.018); 0.004	0.018(0.010–0.026); 0.0001		
Disease duration	−0.82(−0.16–0.002); 0.055					
RBDQ				0.029(0.002–0.057); 0.037		
LEDD						
UPDRS-III						
MOCA						
UPSIT						
NMSQ						

^*^- significance driven by iPD; ^- significance driven by *LRRK2*-PD.

RBDQ- REM sleep Behavior Disorder Questionnaire, LEDD- LevoDopa Equivalent Daily Dose, UPDRS- Unified Parkinson’s Disease Rating Scale part III, MoCA – Montreal Cognitive Assessment, UPSIT- University of Pennsylvania Smell Identification Test, NMSQ- Non-Motor Symptoms questionnaire.

Seventy PD patients (32.7%) in our cohort had MS. This group was older (69.32 ± 8.14 vs. 63.66 ± 10.54; p < 0.001) and had a higher age of diagnosis (66.41 ± 8.42 vs. 60.59 ± 10.19; p < 0.001) but did not differ in any other disease or genetic characteristics.

One hundred ninety-six healthy non *GBA*-*LRRK2*-carriers were compared with 55 *LRRK2*-NMC and 97 *GBA*-NMC (Tables 4 and 5).

**Table 4:** Non-manifesting carriers’ characteristics.

	Control	*LRRK2*-NMC	*GBA*-NMC	Significance
#	196	55	97	
Age (y)	54.57 (11.32)	52.89 (10.58)	54.27 (9.59)	0.517
Sex m/f	89/107	28/27	36/61	0.377
Orthostatic Hypotension (%)	7%	10.09%	10.30%	0.516
MDS-UPDRS	5.46 (4.35)	5.69 (4.31)	6.06 (6.79)	0.449
MoCA	26.46 (2.77)	26.43 (2.74)	25.86 (3.27)	0.308
UPSIT	30.39 (6.82)	31.42 (4.93)	29.45 (6.89)	0.163
RBDQ	1.75 (1.67)	1.42 (1.57)	1.98 (1.96)	0.281
NMSQ	3.39 (3.48)	2.75 (2.78)	3.35 (3.58)	0.699
SCOPA-AUT	8.42 (7.22)	7.98 (5.96)	8.47 (7.90)	0.252
BDI	4.16 (5.26)	3.61 (4.70)	4.22 (5.65)	0.304
Probability of prodromal PD (%)	10.95 (20.17)	33.83 (31.54)^@^	16.09 (25.70)	0.001

NMC- Non-Manifesting Carriers, m-male, f-female, MDS-UPDRS- Movement Disorder Society - Unified Parkinson’s Disease Rating Scale, MoCA – Montreal Cognitive Assessment, UPSIT- University of Pennsylvania Smell Identification Test, RBDQ- REM sleep Behavior Disorder Questionnaire, NMSQ- Non-Motor Symptoms questionnaire, SCOPA-AUT- Scale of Outcome in Parkinson’s Disease – Autonomic Dysfunction, BDI- Beck Depression Inventory.

^@^Differences between *LRRK2*-NMC and *GBA*-NMC, control.

**Table 5:** Non-manifesting carriers’ Metabolic Syndrome components.

	Control	*LRRK2*-NMC	*GBA*-NMC	Significance
#	196	55	97	
HDL (mg/Dl)	56.45 (15.76)	56.44 (14.47)	57.84 (17.16)	0.694
Triglycerides (mg/Dl)	136.89 (75.66)	146.36 (105.63)^@^	142.00 (75.01)	0.014*
HbA1c (%)	5.49 (0.48)	5.45 (0.43)	5.44 (0.44)	0.683
BMI (kg/m^2^)	26.36 (4.78)	26.15 (4.26)	26.66 (4.69)	0.785
Hypo-HDL (%)	18.97%	23.63%	24.21%	0.525
Hypertriglyceridemia (%)	43.58%	56.36%	49.47%	0.619
Prediabetes (%)	30.25%	21.81%	25.26%	0.393
Hypertension (%)	65.12%	58.18%	58.90%	0.469
Obesity (BMI > 30) (%)	20.51%	14.54%	27.36%	0.163

NMC- Non-Manifesting Carriers, HDL- High Density Lipoprotein, HbA1c – Hemoglobin A1C, BMI- Body Mass Index. ^*^Significant after correcting for multiple comparisons with Bonferroni adjustment.

^@^Differences between *LRRK2*-NMC and *GBA*-NMC, control.

*LRRK2*-NMC had higher triglyceride levels (p = 0.014) which were correlated with SCOPA-AUT (r = 0.410, p = 0.003) and with the probability score (r = 0.322, p = 0.019). HbA1c was also correlated with the probability score (r = 0.532, p = 0.010) among this group.

Among the NMC, the number of metabolic risk factors was associated with sex and age but not with genetic status in the linear regression model which was constructed in order to estimate the relationship between MS and its’ components (as dependent variables) and genotype, sex, age, probability score, RBDQ, UPDRS-III, MoCA, UPSIT and NMSQ (independent variables). As with PD, no association between participants’ characteristics, obesity and HDL levels were detected. Prediabetes was associated with age and UPDRS-III scores accounting for 38.8% of the variance. Hypertension was associated with age and male sex accounting for 35.7% of the variance. Hypertriglyceridemia was associated with age and male sex accounting for 41.9% of the variance (Table 6).

**Table 6:** Linear regression Models for metabolic factors in unaffected carriers. B(95%CI) and p values are presented for measures found significant in the model.

	Number of metabolic factors	Prediabetes	Hypertension	Hypertriglyceridemia	Low HDL	Obesity
Model	F = 7.137; p < 0.001	F = 5.857; p < 0.0001	F = 0.481; p < 0.001	F = 7.030; p < 0.0001	F = 0.619; p = 0.781	F = 1.148; p = 0.329
Genotype						
Sex	−0.459(−0.745–0.173); 0.002	−0.095(−0.194–0.003); 0.058	−0.171(−0.279–0.064); 0.002	−0.140(−0.248–0.032); 0.002		
Age	0.048(0.033–0.063); 0.0001	0.015(0.010–0.020); 0.0001	0.014(0.008–0.019); 0.0001	0.016(0.011–0.022); 0.0001		
Probability						
RBDQ						
UPDRS-III		−0.042(−0.077–0.007); 0.018				
MOCA		−0.017(−0.035–0.001); 0.061				
UPSIT						
NMSQ						

RBDQ- REM sleep Behavior Disorder Questionnaire, UPDRS- Unified Parkinson’s Disease Rating Scale part III, MoCA- Montreal Cognitive Assessment, UPSIT- University of Pennsylvania Smell Identification Test, NMSQ- Non-Motor Symptoms questionnaire.

Ten participants (3%) had a likelihood ratio for converting to PD (probability score) higher than 80% (4 non-carriers, 3 *GBA*-NMC, 3 *LRRK2*-NMC) placing them under the category of possible prodromal PD^1^. Most of the cohort (57.8%) had a probability score of less than 2%. Splitting the non-manifesting groups based on the probability score resulted in 25 NMC with a score above 50 and 323 below it. Between group differences were noted in hypertriglyceridemia (43.7% vs 72.0%, p < 0.005), prediabetes (26.7% vs. 48.0%, p = 0.023), but not in hypertension (60.8% vs. 76.0%, p = 0.095), obesity (20.1% vs 32.0%, p = 0.125), low HDL levels (21.6% vs. 24.0%, p = 0.470) or the number of metabolic components (1.72 vs. 2.52, p = 0.087).

Ninety-Four (27.2%) non-manifesting participants had MS. This group was significantly older (59.3 ± 11.2 vs. 52.4 ± 9.9; p < 0.001), had less years of education (16.6 ± 2.7 vs. 17.5 ± 2.7; p = 0.015), lower MoCA scores (25.5 ± 3.7 vs. 26.6 ± 2.5; p = 0.007) and higher SCOPA-AUT scores (10.0 ± 8.9 vs. 7.8 ± 6.4; p = 0.039), however, participants with MS did not have a higher risk to develop PD based on the probability score (p > 0.170).

## Discussion

We used an enriched cohort of *GBA* and *LRRK2* PD patients and non-manifesting carriers in order to determine the association between the metabolic syndrome and its’ components with PD phenotype and study the influence of the metabolic syndrome on the prevalence of prodromal features of PD among individuals at risk for future disease. We did not detect a worse motor or non-motor profile among PD patients who had a concurrent diagnosis of MS. This finding was strengthened by the fact that both *LRRK2*-PD and *GBA*-PD patients were more advanced in their disease state with higher LEDD compared with iPD. The increase of metabolic components burden was not associated with a higher probability scores for PD among NMC and does not seem to influence the phenptype of G2019S *LRRK2-* PD or  *GBA*-PD.

*LRRK2*-PD had elevated levels of triglycerides and higher rates of prediabetes, with no relationships to clinical phenotype. Among *LRRK2*-NMC higher levels of triglycerides were also detected. Both higher triglycerides and HbA1c were positively correlated with the probability score of prodromal PD among *LRRK2*-NMC. The MDS task force specified probable prodromal PD as a likelihood of 80%, but they also allowed, in specific research settings a more lenient cutoff^1^.

Among NMC with high probability for future development of PD (>50%), disregarding genetic status, hypertriglyceridemia and the presence of prediabetes were detected, attesting to a possible contribution of these components of the MS to PD pathogenesis.

MS effect, as is that of its’ components, on risk for developing PD and severity of PD phenotype is still contested^15,24^, with conflicting reports regarding BMI^25–27^ and hypertension^22,28^. To this extent, our findings do not support a role for obesity on disease phenotype.

The association between diabetes mellitus (DM) and risk of PD is also still contested^29,30^ but a meta-analysis reported increased pooled relative risk of developing PD after DM^17^. DM and PD are associated with inflammation, oxidative stress and mitochondrial dysfunction, while glycation of alpha synuclein has been suggested to promote aggregation of this protein^31^. Prediabetic PD patients were found to have worse motor symptoms, faster motor progression and more severe cognitive decline compared to normo-glycemic PD patients^29,32^. 57% of our cohort of PD patients was prediabetic within range of previous studies^29^. *LRRK2*-PD had higher rates of prediabetes compared with both iPD and *GBA*-PD with no clinical impediment. NMC with high probability for future PD had higher rates of prediabetes as well, suggesting a possible role in the pathogenesis of PD.

High levels of triglycerides and LDL-C have been associated with decreased risk of PD among a cohort of Israeli adults^33^ as among other cohorts as well^15,34^. The phosphorylation of Rab8a by *LRRK2* has been shown to alter the ability of lipid storage in PD, while its’ significance on total lipid levels and triglycerides has yet to be determined^35^. We observed higher levels of triglycerides among *LRRK2*-PD, *LRRK2*-NMC and non-genetic controls with increased probability rates for future development of PD; however, the clinical significance will require future corroborating studies.

Gaucher disease has been associated with insulin resistance^36^ and increased hepatic glucose output^37^, with lower levels of LDL and HDL cholesterol and higher levels of triglycerides^38^. We did not find increased prevalence of components of MS among *GBA*-PD nor could we detect any associations between these and the risk to develop PD among *GBA*-NMC. Based on our cross-sectional observational data we cannot suggest an effect of MS or its sub-components on the disease process in *GBA*-PD.

The strengths of this study include a relatively large and well-defined genetic cohort. Additionally, the prospective cohort design enables the exploration of MS and its components without relying on self-report of current medical conditions but rather on medication lists and laboratory results. Limitations include lack of information regarding smoking, alcohol consumption, and the timing of appearance of MS components relative to onset of PD. We did not assess the response of the different components of MS to the medical regiment but coded all participants who took MS component-related medications as positive for the component. Previous use of any MS related medication was not assessed. Blood samples were not collected after a night fast; however, this was uniform for all participants and is gaining acceptability in clinical studies^39^, nevertheless this might have caused bias in this research setting. The MDS-prodromal score incorporates genetic status into the model giving *LRRK2* 25 points and *GBA* between 2–10 points depending on the severity of the mutation^1^, thus *LRRK2*-NMC inherently have higher probability scores. This study collected data from both patients with PD and their first degree relatives with and without mutations in designated genes however; shared genetic background goes beyond mutations in the *LRRK2* and *GBA* genes and could potentially influence our results.

Despite these limitations, the findings contribute to our knowledge of MS and its relation to PD. From a clinical perspective, MS is modifiable; hence understanding its impact on the risk of developing PD and the severity of PD is important to future personalized medicine. The importance of this topic, therefore, warrants further research.

## Methods

This study evaluated cross-sectional demographic, laboratory and questionnaire data from subjects who participated in the BEAT-PD study (TLV-0204–16), a collaborative venture between Biogen and the Tel-Aviv Medical Center, which set out to characterize *LRRK2* and *GBA* PD as well as NMC of these mutations. Patients were recruited consecutively if they were AJ, diagnosed with PD by a movement disorders specialist based on the UK brain bank criteria and were at Hoehn and Yahr stages 1–2. Patients were excluded if they had additional neurological or psychiatric disorders, a malignancy or were HIV, HBV or HCV positive. In addition, first degree relatives of patients with PD were recruited to this study if they were above the age of 40, were not diagnosed with PD and did not have a malignancy. Controls were invited to participants if they did not have PD and did not have a malignancy. The study was approved by the local ethical committee of the Tel-Aviv Medical Center, with all participants providing informed consent prior to participation and all methods performed in accordance with the relevant guidelines and regulations.

### Procedure

All participants underwent genetic testing for the G2019S mutation in the *LRRK2* gene and for the common AJ *GBA* mutations as described previously^40^ and were separated based on genetic status. Only heterozygote carriers were included in this study with dual mutation carriers and homozygote carriers excluded.

Disease severity was assessed using the MDS-Unified Parkinson’s Disease Rating Scale (MDS-UPDRS) during ON medication^41^. The Montreal Cognitive Assessment (MoCA) was used to assess global cognitive functions;^42^ mood was assessed using the Beck Depression Inventory (BDI)^43^. The Non-Motor Symptoms Questionnaire (NMSQ)^44^, Scale of Autonomic Function in PD (SCOPA-AUT)^45^ and the REM sleep Behavior Disorder Questionnaire (RBDQ)^46^ were collected. Olfaction was tested using the University of Pennsylvania Smell Identification Test (UPSIT)^47^. These measures were used to calculate the probability for prodromal PD (Likelihood Ratio Score) for all participants without a diagnosis of PD that were above the age of 50, based on the Movement Disorders task force guidelines^1^. This measure has been validated by our group as by others and is updated based on relevant studies^48–50^ Each non-PD subject was allocating a ratio between 0–100% for risk for future development of PD.

Blood pressure was measured in the supine and standing position with orthostatic hypotension categorized as a drop of 20 mm HG in systolic or 10 mm HG diastolic pressure after 5 minutes.

Demographic data on weight, height and full medication list was collected and Levodopa equivalent daily dose (LEDD) was calculated^51^. Medications were separated into the following groups: anti-hypertensive, lipid lowering and anti-glycemic. Blood samples were collected and assessed for HbA1c, triglycerides and HDL cholesterol.

MS was diagnosed if at least three of the following five components were present: Hypertension- blood pressure above 130/85 mm HG in any position, or use of anti-hypertensive medication; Prediabetes- HbA1c above 5.7% or use of anti-glycemic medication; Obesity- if BMI > 30 kg/m^2^; Hypertriglyceridemia- triglycerides> 150 mg/dl or use of lipid lowering medications and Low HDL- 40 mg/dl for men and 50 mg/dl for women or use of lipid lowering medications^9,11,52^.

### Statistical analysis

Descriptive statistics (means and standard deviations (SD) for continuous variables, percent for categorical variables) were computed for all measures. The analysis was performed in a stepwise manner, first we evaluated differences between groups in all collected measures using mixed models (general linear) based on disease status: differences between PD patients based on genetic status and separately differences within the unaffected cohort based on genetic status. The analysis was adjusted for age and sex in both cohorts. For patients with PD, analysis was also adjusted for disease duration and LEDD. Measures that were significantly different between genetic groups within each cohort were then explored for their association with PD symptoms and signs using Pearson correlation coefficient. Differences in the prevalence of MS between each group within each cohort were calculated using chi square tests (χ^2^). In the next step, multiple linear regression models were constructed to estimate the relationship between MS and its’ components (as dependent variables) and genotype, sex, age, RBDQ, UPDRS-III, MoCA, UPSIT and NMSQ (independent variables). For the PD group the model also included disease duration and LEDD, while for subjects without PD, the association to the probability of prodromal PD score was also explored. Significance was determined for at p < 0.05 for descriptive measures and corrected for multiple comparisons using Bonferroni adjustment for the metabolic components. Statistical analysis was performed using SPSS (SPSS version 22, Chicago. IL, USA).

## Data Availability

Anonymized data will be made available on request.
